# Protection of the receptor binding domain (RBD) dimer against SARS-CoV-2 and its variants

**DOI:** 10.1128/jvi.01279-23

**Published:** 2023-10-16

**Authors:** Yan Wu, Jian Shi, Xiaoxue He, Jia Lu, Xiao Gao, Xuerui Zhu, Xinlan Chen, Man Zhang, Lijuan Fang, Jing Zhang, Zhiming Yuan, Gengfu Xiao, Pengfei Zhou, Xiaoyan Pan

**Affiliations:** 1 State Key Laboratory of Virology, Wuhan Institute of Virology, Chinese Academy of Sciences, Wuhan, China; 2 Wuhan YZY Biopharma Co., Ltd., Wuhan, China; 3 University of the Chinese Academy of Sciences, Beijing, China; 4 Center for Biosafety Mega-Science, Wuhan Institute of Virology, Chinese Academy of Sciences, Wuhan, China; Loyola University Chicago, Maywood, Illinois, USA

**Keywords:** SARS-CoV-2, receptor-binding domain, homodimer, Omicron XBB.1.16, cross-protection

## Abstract

The severe acute respiratory syndrome coronavirus 2 (SARS-CoV-2) recombinant protein vaccines have been widely used in the real world and shown good protective effects. A vaccine prepared from the ancestral SARS-CoV-2 receptor-binding domain (RBD) homodimer was previously made a candidate in view of its effectiveness in rodents and nonhuman primates. Here, we report that the RBD homodimers of ancestral SARS-CoV-2 as well as the variant RBD dimers from the Beta, Delta, Lambda, Omicron, and Omicron sublineages, which were rapidly prepared using our universal dimeric protein platform, elicit both strong immunogenicity and good protection *in vivo*. The ancestral RBD vaccine was verified to provide cross-protection against the SARS-CoV-2 Delta variant from lethal challenge. A heterogeneous booster with Omicron BA.1 dimeric RBD vaccine based on a two-dose ancestral vaccine prime reduced the viral loads in Omicron BA.1 virus-challenged animals. In addition, vaccines prepared from dimeric Omicron XBB.1.5 RBD completely protected the mice from lethal challenge by Omicron XBB.1.16 and reduced the viral infection in the respiratory tract of Syrian hamsters. Thus, RBD homodimer vaccines can confer good protection against SARS-CoV-2 and its variants when used in homogeneous or heterogeneous boosting schemes.

**IMPORTANCE:**

Severe acute respiratory syndrome coronavirus 2 (SARS-CoV-2) variants achieved immune escape and became less virulent and easily transmissible through rapid mutation in the spike protein, thus the efficacy of vaccines on the market or in development continues to be challenged. Updating the vaccine, exploring compromise vaccination strategies, and evaluating the efficacy of candidate vaccines for the emerging variants in a timely manner are important to combat complex and volatile SARS-CoV-2. This study reports that vaccines prepared from the dimeric receptor-binding domain (RBD) recombinant protein, which can be quickly produced using a mature and stable process platform, had both good immunogenicity and protection *in vivo* and could completely protect rodents from lethal challenge by SARS-CoV-2 and its variants, including the emerging Omicron XBB.1.16, highlighting the value of dimeric recombinant vaccines in the post-COVID-19 era.

## INTRODUCTION

Following the global outbreak of severe acute respiratory syndrome coronavirus 2 (SARS-CoV-2) at the beginning of 2020 (1, 2), SARS-CoV-2 has successively evolved into five dominant variants of concern, including Alpha, Beta, Gamma, Delta, and Omicron, and eight dominant variants were monitored during the past 3 years (Epsilon, Zeta, Eta, Theta, Lota, Kappa, Lambda, and Mu). From 2022, the dominant circulating variant was Omicron, including B.1.1.529 and its sublineages BA.1, BA.1.1, BA.2, BA.3, BA.4/5, BQ.1, BF.7, XBB.1.5, XBB.1.16, EG.5, and so on (https://www.who.int/activities/tracking-SARS-CoV-2-variants). Due to the interaction between viral evolution, such as viral variation and combination, and human interventions, including human activities, physical quarantine, usage of personal protective equipment, vaccine inoculation, and therapeutic use, mutations frequently occurred in the SARS-CoV-2 spike (S) protein and especially in the receptor-binding domain (RBD) (3). Mutations that influence transmissibility, virulence, or immunity are of great concern to both scientists and the public (4). For instance, D614G in the S protein enhanced the infectivity, competitive fitness, and transmission of the SARS-CoV-2 Alpha variant (5 – 7); K417N, E484K, and N501Y in the S protein of the Beta variant increased the binding affinity for the ACE2 receptor, thus increasing the risk of transmission and reducing neutralization (8 – 10); and both K417N and E484A were predicted to have an overwhelmingly disruptive effect, making the Omicron variant more likely to cause breakthrough infections (11, 12).

To address this unprecedented, complex, and volatile epidemic, researchers, as well as manufacturers, spared no effort in developing vaccines to contain the spread of the epidemic as quickly as possible (13, 14). Fortunately, dozens of COVID-19 vaccines, including inactivated vaccines, recombinant protein vaccines, mRNA vaccines, and viral vector vaccines were developed and proved to effectively reduce the fatality rate or severe disease rate in clinical trials (15). Nevertheless, whether the existing vaccines on the market or in development can provide cross-protection against the circulating and emerging SARS-CoV-2 variants is unclear (13). Because vaccine development usually lags behind the viral emergence and the pace of vaccine development is often slower than the pace of viral evolution, new strategies to design broad-spectrum vaccines that perhaps have some foresight for emerging SARS-CoV-2 variants are emerging (16, 17). In addition, some compromise solutions with feasibility and practicality, such as new immunogen designations, immunogen combinations, and homogeneous or heterogeneous boosting, which aim to achieve high neutralizing titers or provide diverse epitopes to enable cross-protection and reduce the breakthrough infections, matter equally (18 – 21).

In our previous study, a homodimer protein strategy based on our dimeric protein platform was adopted to develop a SARS-CoV-2 vaccine that was characterized by high quality, low cost, strong immunogenicity, and good protection (22, 23). Using an Fc tag at the C-terminus, ancestral SARS-CoV-2 RBD homodimers were able to form through a pair of disulfide bonds, and then the protein was converted into a recombinant protein without the tag by utilizing a thrombin cleavage site at the C-terminus of the RBD, which is a completely different method from the tandem strategy employed by ZF2001 (24). Using this dimeric protein platform, the RBD homodimer vaccine was rapidly prepared and entered a phase II clinical trial. Here, we prepared several variant RBD homodimers from SARS-CoV-2 Beta, Delta, Lambda, and Omicron variants as candidate vaccines using the same strategy and evaluated the efficacy of the updated vaccines against an emerging Omicron sublineage. This study provides a universal strategy for dimeric RBD vaccine development and highlights the reliability and feasibility of dimeric RBD vaccines against SARS-CoV-2.

## RESULTS

### Preparation and characterization of RBD homodimers

**Fig 1:**
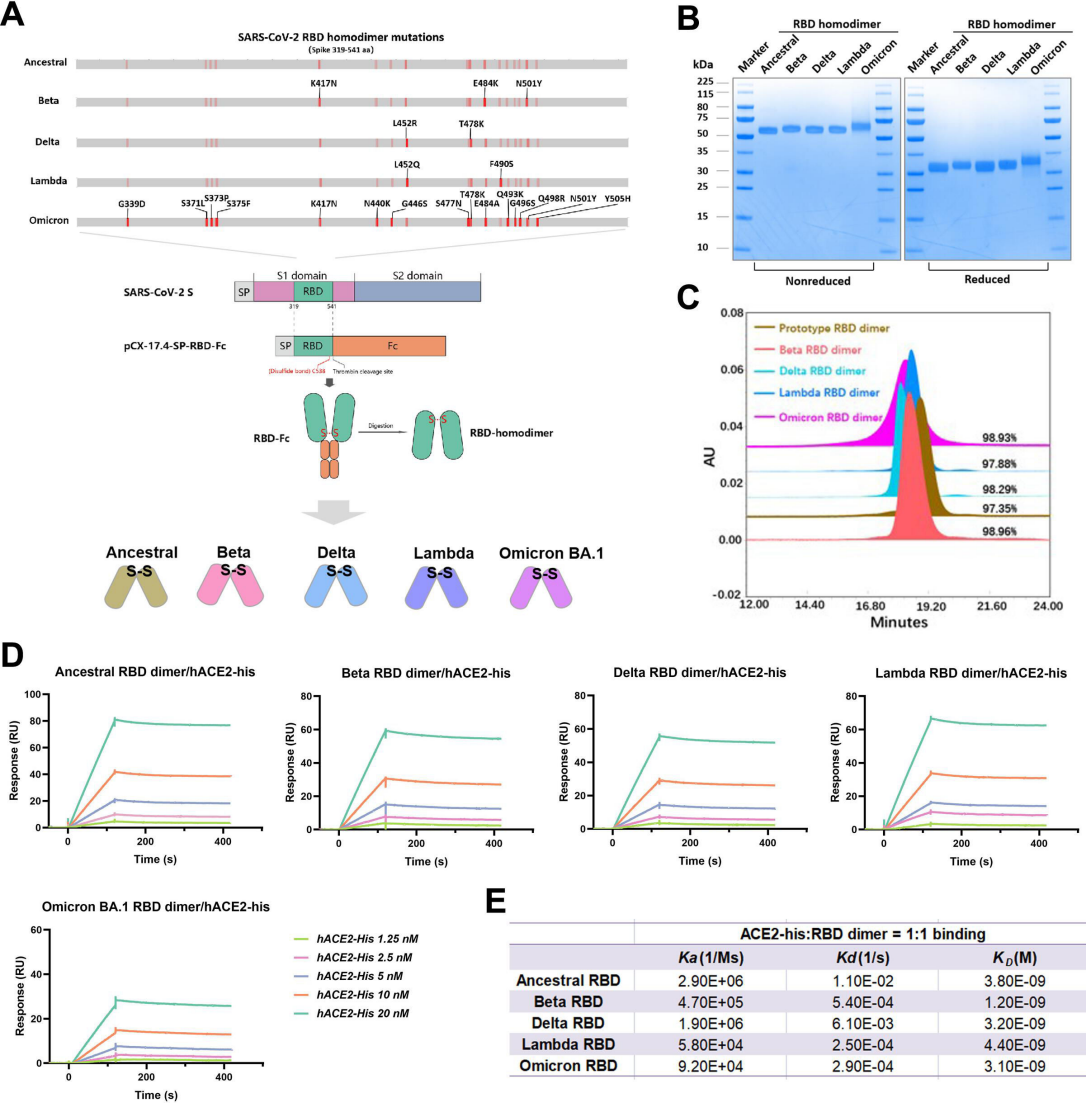
Preparation and characterization of RBD dimers. (**A**) Mutations in the ancestral, Beta, Delta, Lambda, and Omicron BA.1 RBDs (amino acids 319–541) and a flow chart of the preparation of RBD dimers. (**B**) Analysis of the five RBD dimers by reduced or nonreduced SDS-PAGE and Coomassie brilliant blue staining. (**C**) Analysis of the five RBD dimers by size-exclusion chromatography; the corresponding purities are indicated in the graph. (**D and E**) The binding affinity of five RBD dimers to hACE2-his was analyzed and is listed in the table. The corresponding *K_D_* s were calculated using BIAcore T200 Evaluation 3.0 software with the “1:1 binding” model as the curve fitting method.

The genetic sequence of the SARS-CoV-2 S protein has undergone many mutations and deletions during the evolution from the ancestral virus to the Omicron variant. The RBD, which is the active center of the S protein, mediates the binding of viruses to host receptors and has the highest mutation frequency. The Beta, Delta, and Lambda RBDs had two or three mutations in comparison to the ancestral RBD, while 15 mutations occurred in the Omicron RBD (Fig. 1A). Using our universal dimeric protein platform (22), RBD proteins of ancestral SARS-CoV-2 and its variants including Beta, Delta, Lambda, and Omicron BA.1 were prepared as no-tag homodimers. In brief, the introduction of an Fc tag to the C-terminus of the RBD promoted dimer formation. Then, the tag was removed by thrombin digestion, and naked RBD homodimers linked by disulfide bonds were obtained.

As shown in Fig. 1B, the sizes of the reduced and nonreduced RBD dimers were approximately 30 and 60 kDa, respectively, which further confirmed the type of connection mode inside the dimer. And the small differences in size between the RBDs were due to the glycosylation modification, which was demonstrated by PNGase F assay in Fig. S1. Using size exclusion chromatography (SEC), we confirmed that the purity of the five RBD dimers exceeded 97% (Fig. 1C). Furthermore, we verified the exposure of major antigenic sites of the five RBD dimers by surface plasmon resonance (SPR) assays on the human ACE2 receptor protein. As expected, the five RBD dimers all showed high affinity for ACE2, and the *K_D_* ranged from 1.2 to 4.4 nM (Fig. 1D and E). These data indicated that the ancestral and variant RBD homodimers exhibited good properties, which enabled the preparation of vaccines.

### Immunogenicity of RBD dimers in BALB/c mice

**Fig 2:**
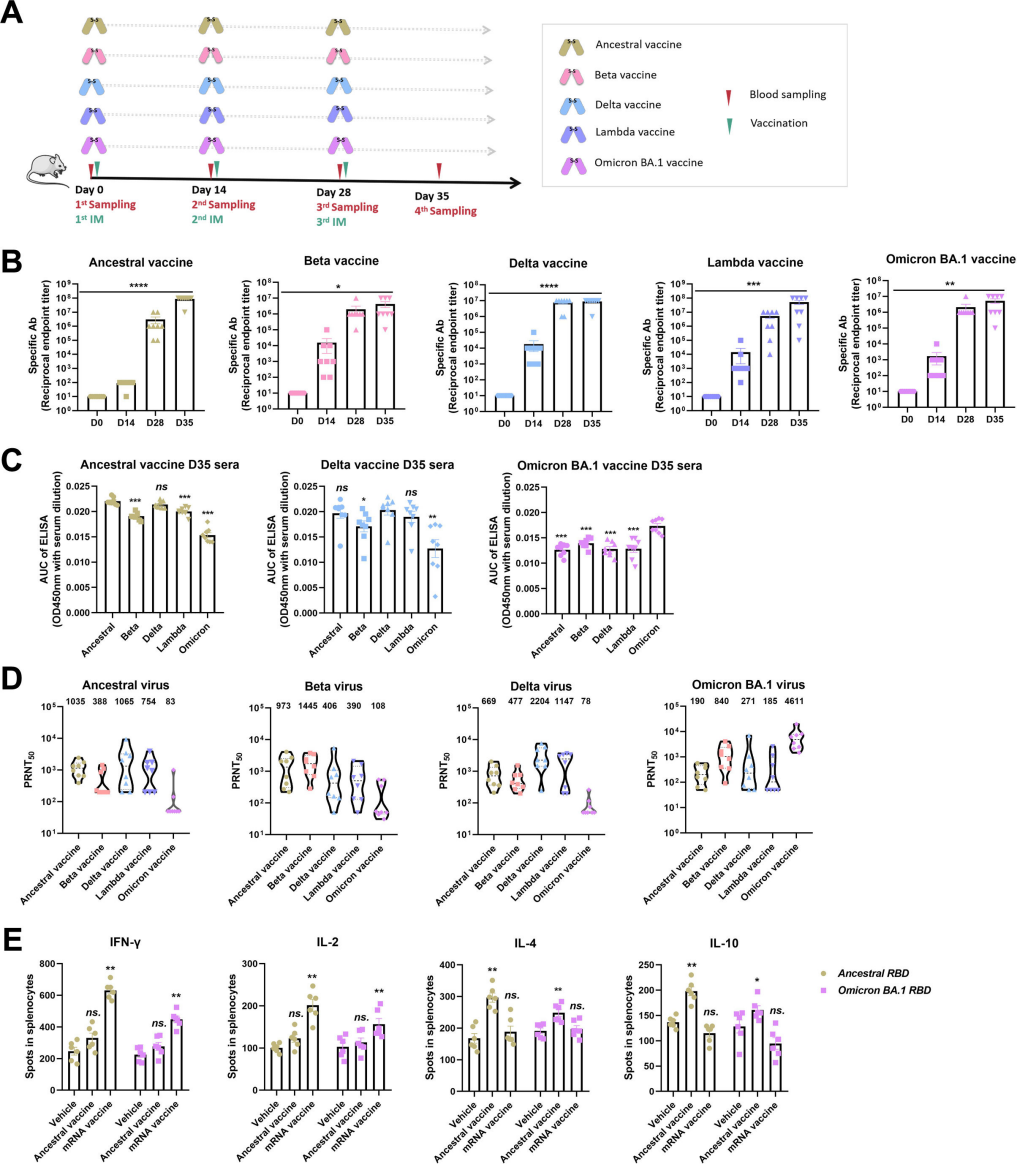
Cross-reactive immune response induced by the five RBD dimer vaccines. (**A**) Vaccination scheme. Six- to eight-week-old female BALB/c mice (*n* = 8 or 6) were vaccinated with ancestral, Beta, Delta, Lambda, and Omicron BA.1 RBD dimers formulated with AL at a dose of 10 µg per mouse on days 0, 14, and 28. Serum samples were obtained from orbital veins 7 or 14 days after each vaccination. (**B**) The titers of specific binding antibodies targeting the corresponding immunogens in the five vaccinated groups were measured by ELISA. (**C**) Cross-binding antibodies in the ancestral, Delta, and Omicron BA.1 vaccine-vaccinated groups targeting the five immunogens were detected by ELISA, and statistical analysis was referred to ancestral, Delta, or Omicron BA.1 antigen, respectively. (**D**) The titers of neutralizing antibodies against the authentic ancestral, Beta, Delta, and Omicron BA.1 viruses in the five vaccinated groups were measured by PRNT. Geometric mean titers calculated from PRNT_50_s are presented on the top of each column. (**E**) Splenocytes from ancestral vaccine-vaccinated BALB/c mice were stimulated with either ancestral or Omicron BA.1 RBD, and Th1/2 cytokines, such as IFN-γ, IL-2, IL-4, and IL-10, were detected by ELISPOT, and AL and ancestral mRNA vaccine were used as controls.

RBD dimers were prepared into ancestral, Beta, Delta, Lambda, and Omicron BA.1 vaccines using a formulation that included an aluminum hydroxide (AL) adjuvant, and the vaccines were tested in BALB/c mice according to a three-shot regimen with 2-week intervals as the schedule presented in Fig. 2A. As detected by enzyme-linked immunosorbent assay (ELISA), the five vaccines all elicited high specific binding antibody titers to the RBDs, especially after the second and third shots, which led to titers of up to ~1:10^6^ and ~1:10^7^, respectively (Fig. 2B). Sera collected from the ancestral, Delta, and Omicron BA.1 vaccine groups after the third shot were used for the detection of cross-binding antibodies against the five RBDs. As shown in Fig. 2C, the binding of serum antibodies from the ancestral or Delta vaccine group to the Omicron BA.1 RBD was obviously weaker than the binding to the other three RBDs. Moreover, the binding of serum antibodies from the Omicron BA.1 vaccine group to ancestral, Beta, Delta, and Lambda RBDs was weaker than the binding to the Omicron BA.1 RBD itself. This finding indicates that a narrow spectrum of cross-reactive antibodies is induced by the Omicron BA.1 RBD.

Furthermore, sera collected from the five groups after the third shot were used for cross-neutralizing antibody detection against ancestral SARS-CoV-2 and its variants through the authentic virus plaque reduction neutralization test (PRNT). As shown in Fig. 2D, the PRNT_50_s of serum antibodies collected from the ancestral, Beta, Delta, and Omicron BA.1 vaccine group against the ancestral viruses varied, exhibiting a trend toward lower levels of antibodies from the Omicron BA.1 vaccine in particular, which was consistent with the previous reports (25, 26). Moreover, the PRNT_50_s of serum antibodies from the ancestral, Beta, and Delta vaccine groups against the Omicron BA.1 virus decreased to varying degrees. Notably, serum antibodies from the Omicron BA.1 vaccine group only showed prominent neutralization of the Omicron BA.1 virus itself and exhibited low cross-neutralizing activity against the ancestral, Beta, and Delta viruses.

In addition, splenocytes from ancestral vaccine-vaccinated mice were stimulated with either ancestral or Omicron BA.1 RBD to observe the CD4^+^ T-cell response detected by enzyme-linked immunospot assay (ELISPOT). In Fig. 2E, in comparison to the ancestral RBD mRNA vaccine, our ancestral RBD dimer vaccine principally triggered a Th2-biased immune response, which was reflected by the significantly increased release of IL-4 and IL-10 rather than IFN-γ or IL-2. Moreover, the release of cytokines stimulated by Omicron BA.1 RBD was weaker than that stimulated by ancestral RBD, which may imply the presence of a small number of conserved epitopes between ancestral and Omicron BA.1 RBD. These results collectively indicated that the RBD dimers perform well in triggering an antibody response, despite the cross-reactive antibody being weak between Omicron and the previous variants as well as the ancestral RBD.

### The ancestral RBD vaccine provided cross-protection against the SARS-CoV-2 Delta variant in a lethal challenge model

**Fig 3:**
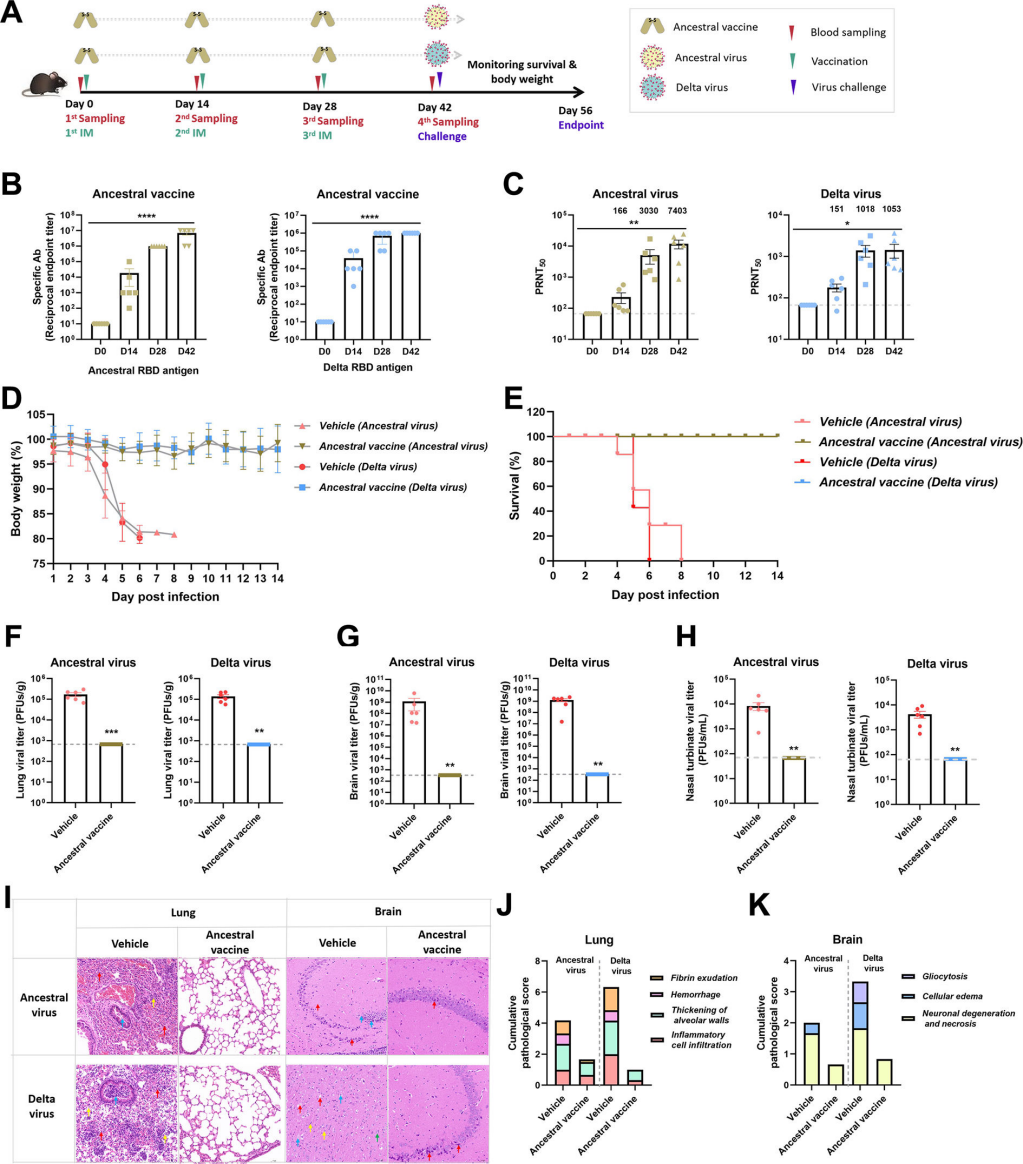
The ancestral RBD vaccine provided cross-protection against the SARS-CoV-2 Delta variant. (**A**) Vaccination and challenge scheme. Six- to eight-week-old K18-hACE2-transgenic C57BL/6J mice (*n* = 6) were vaccinated with ancestral 10 µg RBD vaccine on days 0, 14, and 28. Serum samples were obtained from orbital veins 14 days after each vaccination. SARS-CoV-2 (ancestral and Delta virus, 1 × 10^3^ PFUs) was used to inoculate mice via the nasal cavity. (**B**) The levels of cross-binding antibodies targeting the ancestral and Delta RBDs were measured by ELISA. (**C**) The levels of cross-neutralizing antibodies targeting the ancestral and Delta viruses were measured by PRNT. (**D and E**) Body weight and survival rate were monitored throughout the whole experimental period. (**F–H**) The viral loads in the lung, brain, and nasal turbinate from each mouse at the endpoint were measured by the plaque formation method in Vero E6 cells. (**I**)The lung and brain tissues at the endpoint were analyzed by H&E staining. Representative images from each group are displayed. Arrows in the lung images are used to indicate areas that exhibit fibrin exudation, hemorrhage, alveolar wall thickening, and inflammatory cell infiltration, and arrows in the brain images are used to indicate areas with gliocytosis, cellular edema, and neuronal degeneration necrosis in the hippocampus. (**J and K**) Accumulative pathological scores from different pathological indicators in the lung and brain were calculated from each mouse.

Human ACE2-transgenic C57BL/6J mice were vaccinated with the ancestral RBD vaccine following a regular procedure and challenged with the ancestral SARS-CoV-2 or Delta variant virus as described in Fig. 3A. As in previous experiments, sera were collected after each vaccination to measure the levels of specific or cross-binding antibodies by ELISA and to detect neutralizing antibodies by PRNT. Mice were all challenged with 1 × 10^3^ plaque-forming units (PFUs) of either the ancestral or Delta virus after the third vaccination. Body weight was monitored during the whole experimental period. The lung and brain tissues were dissected for viral RNA load measurement or examined by hematoxylin-eosin (H&E) staining.

Consequently, the ancestral RBD vaccine could elicit cross-binding and cross-neutralizing antibodies in C57BL/6J mice as well as in BALB/c mice after the second and third vaccinations. Specifically, the titer of binding antibodies targeting the ancestral RBD after the third vaccination was ~1:10^7^ and that targeting the Delta RBD dimer was ~1:10^6^ (Fig. 3B). Correspondingly, the titer of neutralizing antibodies targeting the ancestral virus produced by the ancestral vaccine was as high as 1:7,403 after the third vaccination, while that targeting Delta was 1:1,053 (Fig. 3C). These data showed that the levels of cross-binding and neutralizing antibodies against Delta variant declined while remaining high.

After the challenge, body weight loss was sustained until death occurred at 4–8 dpi in the vehicle groups; in contrast, mice in the vaccinated groups all survived within 14 days after the challenge with either the ancestral SARS-CoV-2 or Delta variant (Fig. 3D and E). By analyzing the tissues of mice at the endpoint, we found that the viral loads in the lung and brain as well as nasal turbinate were all under the detection limit, indicating the complete clearance of viruses at 14 dpi (Fig. 3F, G, and H). In line with the viral loads, the lesions in the lung and brain were significantly alleviated and only symptoms that were difficult to eliminate in a short time remained (Fig. 3I, J, and K). These results demonstrated that the ancestral RBD vaccine can provide cross-protection from SARS-CoV-2 Delta variant infection.

### Heterogeneous booster with Omicron BA.1 RBD vaccine can defend against SARS-CoV-2 Omicron BA.1 infection

**Fig 4:**
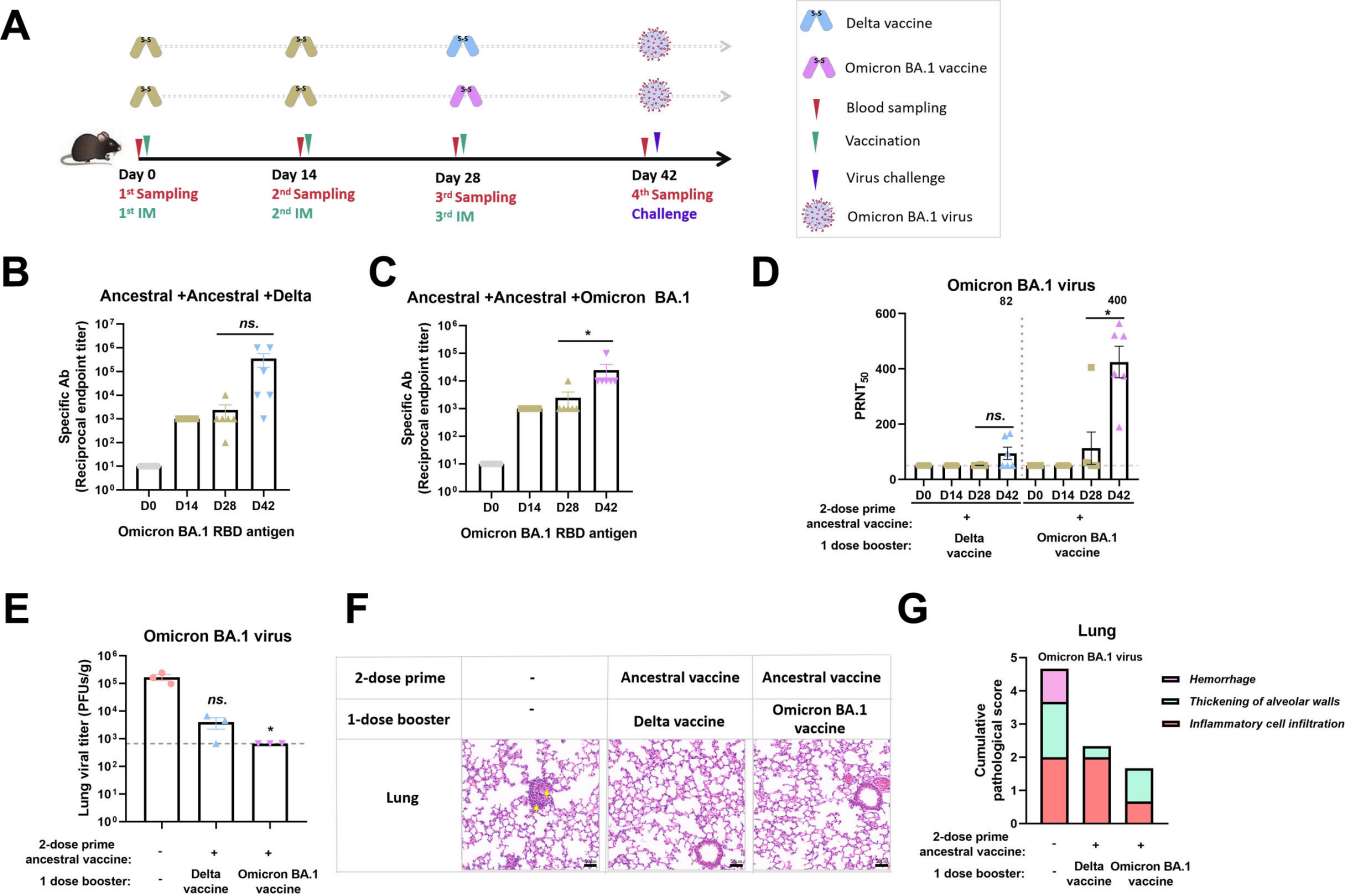
Heterogeneous booster with the Omicron BA.1 vaccine reduced the viral load and pathological injury in SARS-CoV-2 Omicron BA.1-challenged mice. (**A**) Vaccination and challenge scheme. Six- to eight-week-old male K18-hACE2-transgenic C57BL/6J mice (*n* = 6) were vaccinated with 10 µg RBD vaccine on days 0, 14, and 28. Serum samples were obtained from orbital veins 14 days after each vaccination. SARS-CoV-2 Omicron BA.1 viruses (5 × 10^4^ PFUs) were used to inoculate mice via the nasal cavity. (**B and C**) Binding antibodies to the Omicron BA.1 RBD proteins in the Delta or Omicron BA.1 vaccine-boosted groups were detected by ELISA. (**D**) The levels of neutralizing antibodies targeting the Omicron BA.1 virus in the Delta and Omicron vaccine-boosted groups were measured by PRNT. Geometric mean titers are presented in the column indicating the third shot booster. (**E**) Three mice were executed for viral titer detection in the lung at 2 dpi. (**F**) Lung tissues from mice dissected at 2 dpi were analyzed by H&E staining. Representative images from each group are displayed. The arrow indicates an area with hemorrhage, alveolar wall thickening, and inflammatory cell infiltration.

Considering that the ancestral RBD vaccine has difficulty in providing sufficient cross-protection for the Omicron variant, we further investigated the efficacy of an alternative vaccination regimen for defending against Omicron, as presented in Fig. 4A. Based on a two-shot ancestral vaccine prime, a heterogeneous booster with the Omicron BA.1 vaccine could slightly improve the titer of binding antibodies to Omicron BA.1 RBD, while the Delta vaccine could not (Fig. 4B and C). Meanwhile, the booster with the Delta vaccine cloud did not markedly elevate the levels of neutralizing antibodies against the Omicron BA.1 virus, whereas the booster with Omicron BA.1 vaccine cloud did (Fig. 4D).

Upon finishing the vaccination procedure, mice were challenged with 5 × 10^4^ PFUs of Omicron BA.1 virus, and viral load measurement and pathological assessment of the lung were performed. As a result, there were no substantial changes in weight loss within 2 days after the Omicron BA.1 virus challenge (Fig. S2A). The viral load in the lung was approximately 10^5^ PFUs per gram at 2 dpi, and this was reduced by boosting with the Omicron BA.1 vaccine (Fig. 4E). In addition, the lesions in the lung were also obviously relieved by boosting with the Omicron BA.1 vaccine (Fig. 4F and G; Fig. S2B). These results suggested that a heterogeneous booster with Omicron BA.1 RBD vaccine could combat Omicron BA.1 infection *in vivo*.

### The Omicron XBB.1.5 RBD vaccine reduces the infection of Omicron XBB.1.16 in the respiratory tract of Syrian hamsters

**Fig 5:**
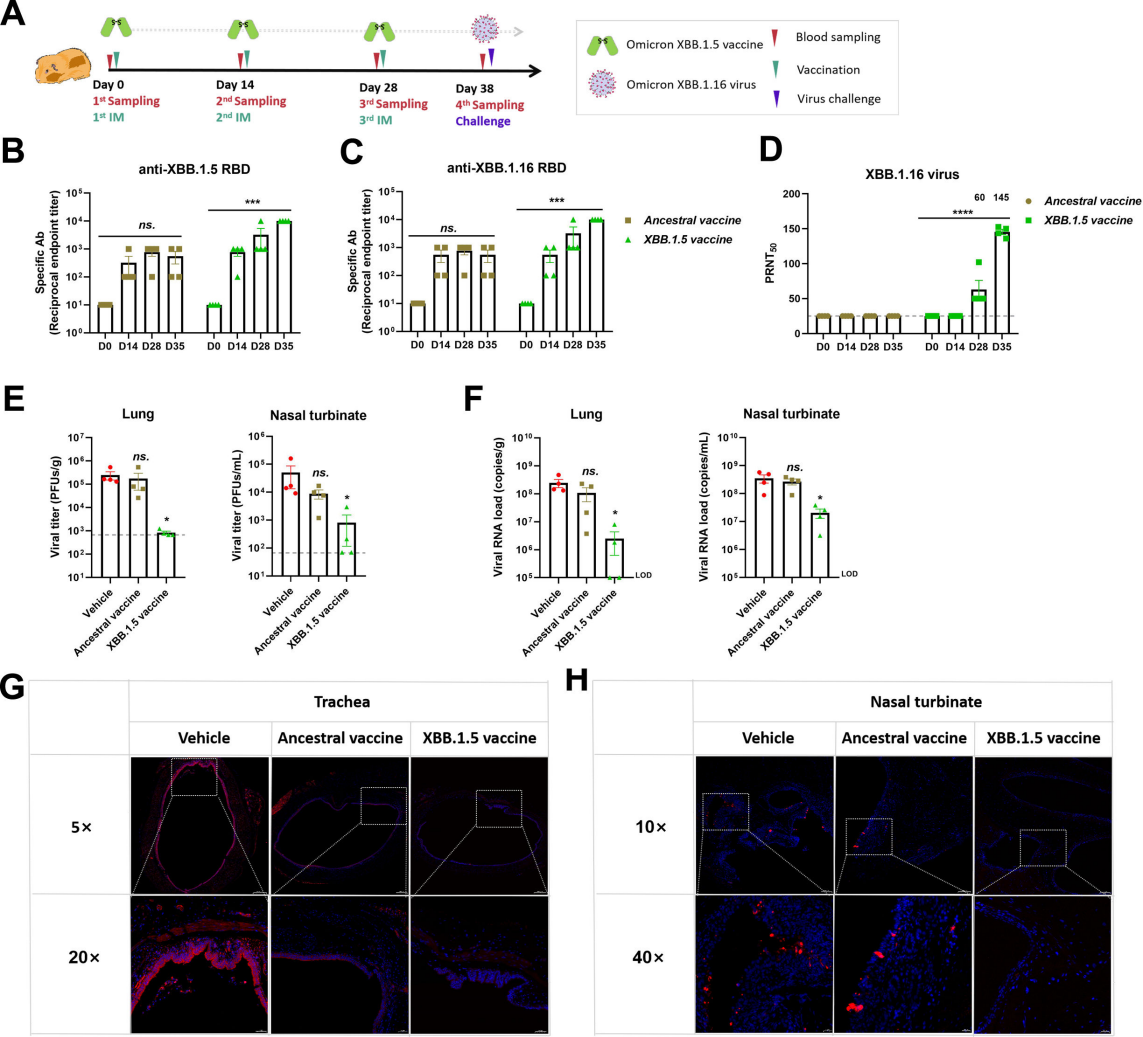
The Omicron XBB.1.5 RBD vaccine reduced Omicron XBB.1.16 infection in Syrian hamsters. (**A**) Vaccination and challenge scheme. Six- to eight-week-old female Syrian hamsters (*n* = 4) were vaccinated with 10 µg Omicron XBB.1.5 RBD dimers formulated with AS03 via IM injection according to a prime-boost-boost protocol with 2-week intervals. The ancestral RBD vaccines formulated with AL and AL only were made as controls. Sera were collected on days 0, 14, 28, and 35. SARS-CoV-2 Omicron XBB.1.16 virus (1 × 10^4^ PFUs) was used to inoculate mice via the nasal cavity on day 35. (**B**) and (**C**) Titers of specific antibodies targeting Omicron XBB.1.5 and XBB.1.16 RBD were measured after each vaccination. (**D**) Titers of neutralizing antibodies against Omicron XBB.1.16 virus were measured, and geometric mean titers are presented on the top of the columns. (**E and F**) The viral titers and copies in the nasal turbinate and lung were measured at dpi 3. (**G and H**) Immunofluorescence analysis of viral antigens in the trachea and nasal turbinate. Sections were stained with antibodies targeting SARS-CoV-2 NP (red), and DAPI was used to stain the nucleus (blue).

The Omicron XBB.1.5 RBD dimer was prepared in the same way as the previous dimers on our dimeric protein platform (Fig. S3). Formulated with the AS03 adjuvant (27), the Omicron XBB.1.5 vaccine was administrated via intramuscular (IM) injection with a regular three-shot regimen in a Syrian hamster model (Fig. 5A). Specific antibodies from the Omicron XBB.1.5 vaccine group for Omicron XBB.1.5 and XBB.1.16 RBD were detected by ELISA after each vaccination, and 1:10^4^ was achieved after the third shot. In contrast, the titers of specific antibodies from the ancestral vaccine group were 1:10^3^ (Fig. 5B and C). In line with that, the titer of neutralizing antibodies against Omicron XBB.1.16 virus from the Omicron XBB.1.5 vaccine group was 1:145 after the third shot, while sera from the ancestral vaccine group had almost no neutralizing effects on Omicron XBB.1.16 virus (Fig. 5D).

After the challenge with Omicron XBB.1.16 virus, slight body weight changes were observed in Syrian hamsters (Fig. S4). At 3 dpi, the viral load in the nasal turbinate and lung peaked at 10^5^ PFUs per milliliter, which was reduced to 10^3^ PFUs per milliliter by the Omicron XBB.1.5 vaccine; moreover, the ancestral vaccine could not reduce the viral loads in either the nasal turbinate or lung and the same results were observed for viral copies (Fig. 5E and F). Since the tissue injury of Syrian hamsters is generally mild, we mainly analyzed the viral infection in the trachea and nasal turbinate. Consistent with the viral loads, the Omicron XBB.1.16 virus infection in the trachea and nasal turbinate was obviously lessened by the Omicron XBB.1.5 vaccine, while this did not occur with the ancestral vaccine (Fig. 5G and H).

### The Omicron XBB.1.5 RBD vaccine provided cross-protection against Omicron XBB.1.16 virus in a lethal infection model

**Fig 6:**
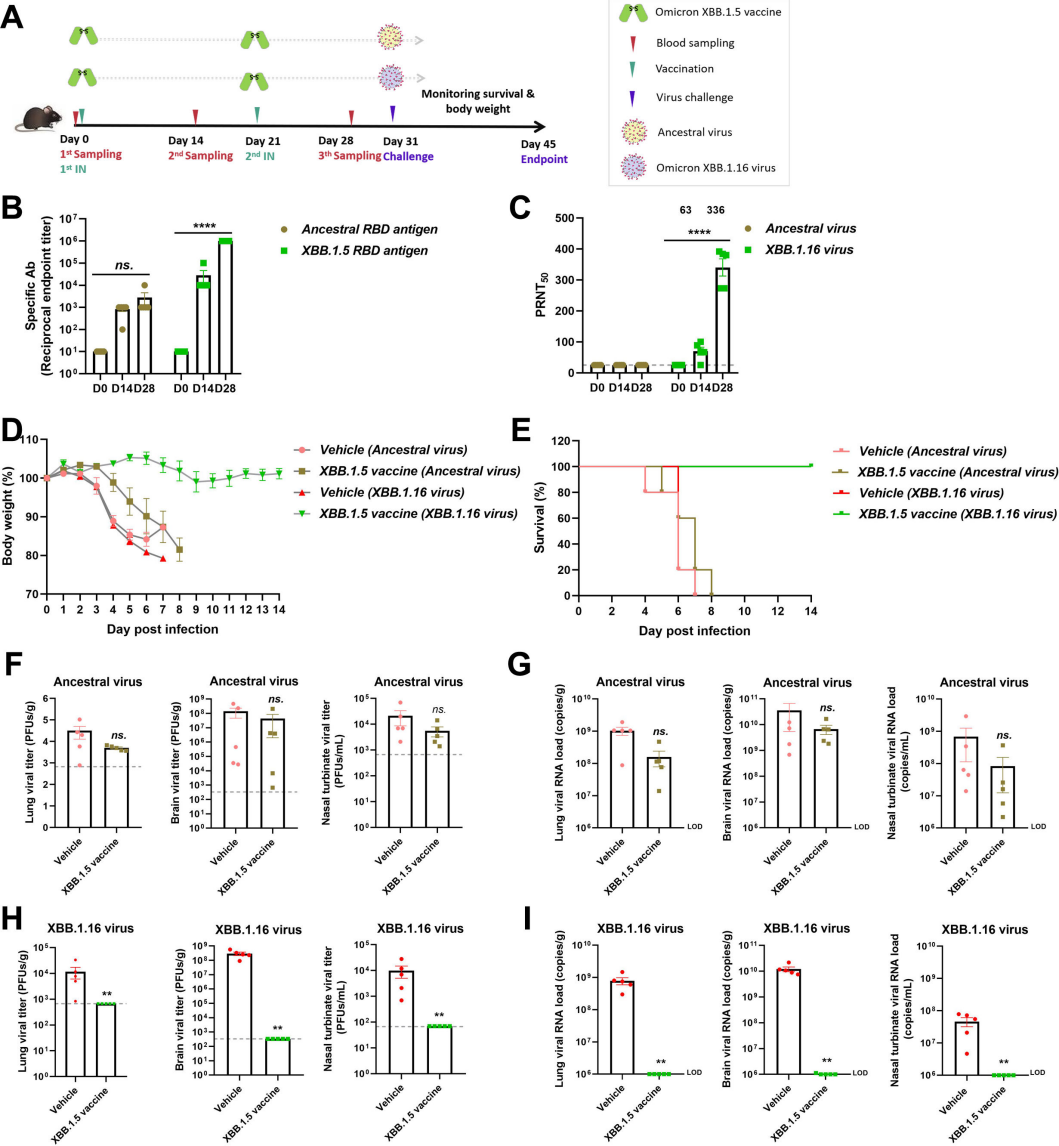
The Omicron XBB.1.5 RBD vaccine protected mice from lethal challenge by Omicron XBB.1.16. (**A**) Vaccination and challenge scheme. Six- to eight-week-old K18-hACE2-transgenic C57BL/6J mice (*n* = 5) were vaccinated intranasally with 10 µg Omicron XBB.1.5 RBD dimers formulated with 2′, 3′-cGAMP on days 0 and 21. Serum samples were obtained from orbital veins on days 14 and 28. SARS-CoV-2 (Omicron XBB.1.16, 1 × 10^4^ PFUs) was used to inoculate mice via the nasal cavity. (**B**) The levels of cross-binding antibodies targeting the ancestral or Omicron XBB.1.5 RBD were measured by ELISA. (**C**) The levels of cross-neutralizing antibodies targeting the ancestral or Omicron XBB.1.16 virus were measured by PRNT. (**D and E**) Body weight and survival rate were monitored throughout the whole experimental period. (**F and G**) The viral copies and titers in the lung, brain, and nasal turbinate from ancestral virus-challenged mice. (**H and I**) The viral copies and titers in the lung, brain, and nasal turbinate from Omicron XBB.1.16 virus-challenged mice.

Formulated with a stimulator of interferon gene agonist, 2′, 3′-cGAMP adjuvant (28), the Omicron XBB.1.5 RBD vaccine was administrated via intranasal (IN) immunization with a two-shot regimen in hACE2-transgenic C57BL/6J mice (Fig. 6A). The titer of Omicron XBB.1.5 RBD-specific antibodies after the second shot reached at 1:10^6^, which was significantly higher than the titer of ancestral RBD-specific antibodies (1:10^4^) (Fig. 6B). From the neutralizing test, the neutralizing titer of the Omicron XBB.1.5 vaccine group was evaluated with the vaccination times and reached at 1:336 after the second shot, while the sera from the Omicron XBB.1.5 vaccine group had no neutralizing effect on the ancestral virus (Fig. 6C).

**Fig 7:**
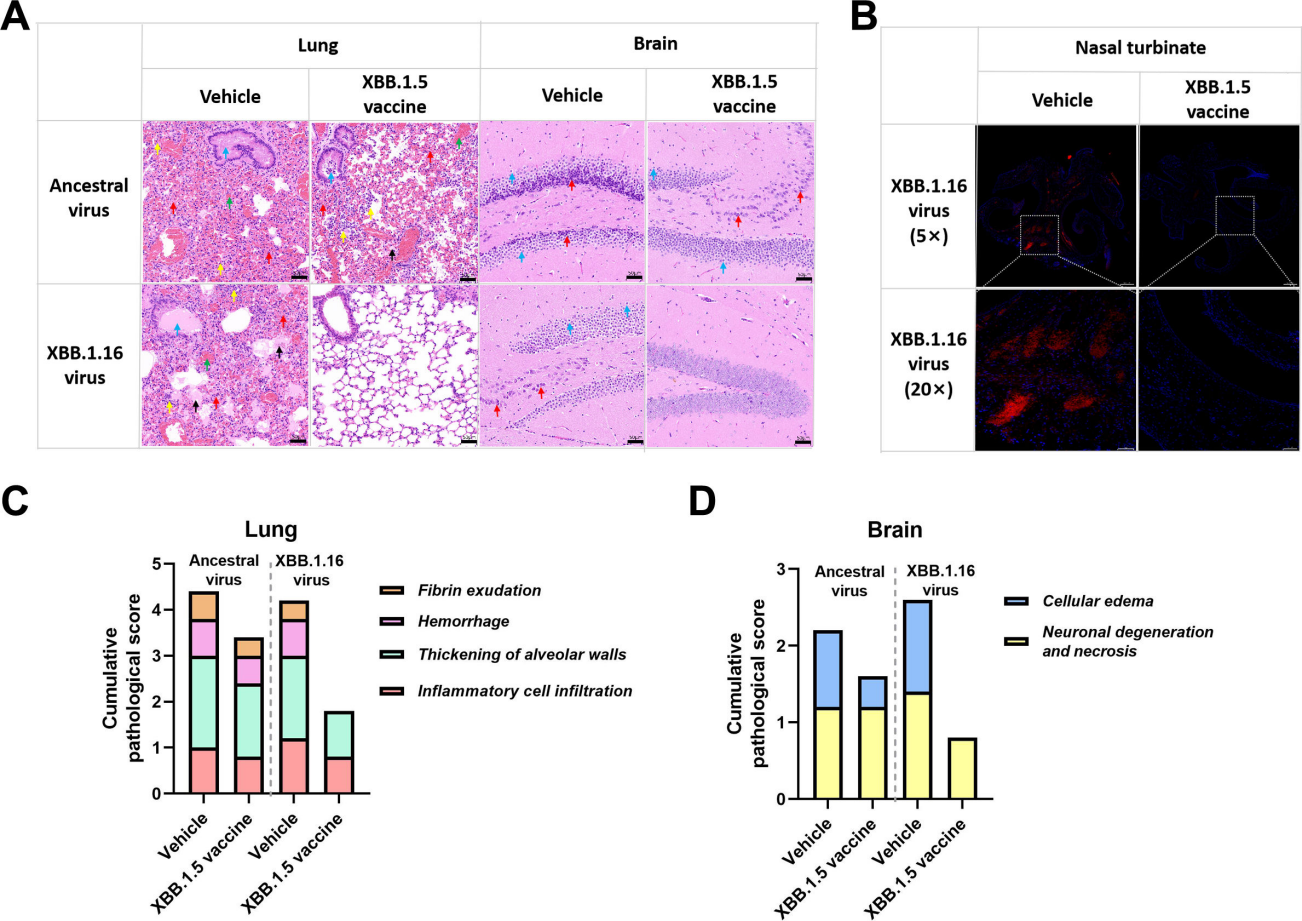
Reduction of pathological damage and infection in Omicron XBB.1.5 RBD-vaccinated mice. (**A**) Lung and brain tissues at the endpoint were analyzed by H&E staining. Representative images from each group are displayed. Arrows in the lung images are used to indicate areas that exhibit fibrin exudation, hemorrhage, alveolar wall thickening, and inflammatory cell infiltration, and arrows in the brain images are used to indicate areas with cellular edema and neuronal degeneration necrosis in the hippocampus. (**B**) Immunofluorescence analysis of Omicron XBB.1.16 infection in the trachea of XBB.1.5 RBD-vaccinated mice. (**C and D**) Accumulative pathological scores from different pathological indicators in the lung and brain were calculated for each mouse.

After the challenge by ancestral or Omicron XBB.1.16 virus, mice continued to lose weight and died at 4–8 dpi in the vehicle groups, while all mice survived in the Omicron XBB.1.5 vaccine-vaccinated group challenged by Omicron XBB.1.16 virus; in contrast, none survived in the ancestral virus-challenged group (one reached the end of mercy) (Fig. 6D and E). From the viral load results, we observed that live virus in the lung, brain, and nasal turbinate was cleared by the Omicron XBB.1.5 vaccine but could not be cleared by the ancestral vaccine, and the viral copies showed similar results (Fig. 6F, G, H, and I). Tissues such as lung, brain, and nasal turbinate at the endpoint were taken for analysis, and the Omicron XBB.1.5 vaccine relieved the lesions in both the lung and the brain (Fig. 7A, C, and D), and significantly reduced the Omicron XBB.1.16 virus infection in the nasal turbinate (Fig. 7B). The results highlight the cross-protection of the Omicron XBB.1.5 RBD vaccine against the emerging SARS-CoV-2 variant, Omicron XBB.1.16.

## DISCUSSION

The ongoing COVID-19 pandemic caused by SARS-CoV-2 has taken a substantial toll and profoundly impacted the human society. As the epidemic spread, the SARS-CoV-2 Omicron variant became the predominant circulating variant since it was first discovered in South Africa in November 2021 (29). In particular, the Omicron variant contains more than 60 mutations in its genome and more than 30 mutations in the S protein (30), implying its strong immune escape, thus highlighting the need for updated vaccines. Our study evaluated the immunogenicity and protection of the dimeric RBD vaccines from variants such as Beta, Delta, Lambda, Omicron BA.1, and XBB.1.5, and confirmed their efficacy in protecting against the emerging Omicron variant in both a lethal challenge model and a mild model. In particular, the AL-adjuvanted ancestral RBD IM vaccine completely protected mice from lethal challenges from the ancestral and Delta viruses; the AS03-adjuvanted Omicron XBB.1.5 RBD IM vaccine significantly reduced Omicron XBB.1.16 virus infection in the respiratory tract; and the 2′, 3′-cGAMP-adjuvanted Omicron XBB.1.5 RBD IN vaccine completely protected mice from lethal challenge from the Omicron XBB.1.16 virus. These results together suggest that the RBD dimer is a good antigen for the SARS-CoV-2 vaccine, regardless of the adjuvants and vaccination routes.

In addition, it is worth mentioning that the ancestral RBD vaccine and Omicron XBB.1.5 vaccine can hardly induce cross-reactivity and especially cross-protection between each other. In our study, ancestral RBD-based vaccine induced limited cross-reactive and -neutralizing antibodies against XBB variants, and four out of five ancestral RBD vaccine-vaccinated mice naturally died after XBB.1.16 challenge, only one reached the end of mercy and was put to death, this means that the rare effective epitopes provided by ancestral RBD, which work in either humoral immune response or cellular immune response, thus cannot provide reliable protection. It is clear that the neutralizing antibody level is a basic and major indicator for evaluating the protection potency (31), rare evidence supports that only cellular immune responses can provide complete protection. However, it is different from that making targets other than the highly diverse RBD, such as the conserved nucleoprotein, as antigen for inducing a cellular immune response, which does provide some protective effect (32). Anyhow, the limited cross-protection between the ancestral and Omicron variants emphasizes the specificity of the emerging variants, which needs ongoing attention, and specific vaccine updates are necessary.

Besides, we demonstrated that heterogeneous boosters may be a practical vaccination regimen, considering that almost all the people have been vaccinated or infected. Since COVID-19 vaccines developed based on ancestral SARS-CoV-2 have a reduced ability or even no ability to neutralize the Omicron variant (33 – 35), some findings suggest that the vaccines are still able to elicit robust T-cell responses against the Omicron variant, and a booster dose of vaccine could recall and expand the preexisting memory immune response against SARS-CoV-2, as well as the *de novo* induction of immune responses, resulting in protection against Omicron infection (36, 37). Therefore, a homogenous or heterogeneous boosting strategy may be able to combat emerging variants such as Omicron, with the advantage that it can improve the titers of neutralizing antibodies as well as provide diverse epitopes that play a key role in both B and T-cell responses (38). These findings may also explain why moderate neutralizing antibodies could still provide some *in vivo* cross-protection using the boosting strategy in our study and clinical trials reported elsewhere (39, 40).

In the past 3 years, numerous SARS-CoV-2 vaccines have been applied to populations around the world and great positive results have been achieved with joint efforts. They are mainly prepared by recombinant protein, mRNA, adenovirus-vectored and virus-inactivated technologies, construction of diverse antigen ingredients and adjuvants, and work by what looks like different immune responses (41). Among them, the recombinant protein vaccines were well-researched both in preclinical and clinical trials, which were based on tandem RBD dimer (42), S timer (43), IFN-α-fused RBD dimer (44), and some other reported antigen designation. The major advantage of the recombinant protein vaccine, including our vaccines reported here, is that they are safe and technically mature, but the strong immunogenicity often depends on suitable adjuvants. Three adjuvants, including aluminum, AS03, and c-GAMP, were employed in our study, despite that they all worked well with the RBD dimers in triggering antibodies and providing protection, a comprehensive comparison of their immune mechanism is worth further study.

The adenovirus-vector vaccine and nucleic acid vaccine are more effective in stimulating the cellular immune response (45) reflected by Th1-type immune cytokines release, while the protein subunit vaccine including the one reported here and the inactivated-virus vaccine mainly induced Th2-type immune responses. Th2-type immune response is helpful for antibody production, while its excessive and persistent activation may lead to tissue damage. In view of that vaccines generally work through short-term local immune stimulation to form long-term immune memory, so this has not occurred with SARS-CoV-2 vaccines including our reported one in the clinical or preclinical trials so far. Definitely, the risk should be monitored along with the virus variation to ensure adequate safety.

After all, this study demonstrated the effectiveness of the RBD dimer as a valid antigen for the SARS-CoV-2 vaccine and the versatility of the dimeric protein platform, while there are small limitations, such as a lack of consistency in comparisons of different adjuvants; thus, which combination of adjuvant and RBD dimer is optimal cannot be determined. Additionally, the BALB/c or C57BL/6J mice seem to respond better to RBD dimer vaccines than Syrian hamsters, which is reflected by both the antibody response and *in vivo* protection; therefore, a suitable model is critical for vaccine evaluation. In addition, the detailed mechanism of different vaccination routes in different antigen-adjuvant combinations is also worthy of further study in the future.

## MATERIALS AND METHODS

### Plasmids, proteins, cell lines, and viruses

SARS-CoV-2 ancestral (GenBank: QHR63260.2), Beta (GenBank: UPE85823.1), Delta (GenBank: UWM25519.1), Lambda (GenBank: QSF03921.1), Omicron BA.1 (GenBank: UVN39829.1), and Omicron XBB.1.5 (GenBank: WEQ09113.1) RBD genes (amino acids 319–541) were codon-optimized for mammalian cell expression and cloned and inserted into the expression plasmid pXC17.4 (LONZA) to obtain RBD-(thrombin site)-Fc constructs. The ancestral, Beta, Delta, Lambda, Omicron BA.1, and Omicron XBB.1.5 RBD homodimers were obtained as previously reported through our dimeric protein platform using a common protocol (22). Specifically, ExpiCHO-S cells were transiently transfected with the plasmid constructs and cultured with serum-free and protein-free medium for 8–12 days. RBD-Fc proteins were purified through protein A agarose affinity chromatography from culture supernatants and digested by thrombin to remove the Fc tag. The RBD proteins were finally obtained by protein A agarose affinity chromatography to remove Fc fragments. Then, the RBD dimers were detected by SEC and reduced or nonreduced SDS-PAGE and formulated with AL (Croda, UK), AS03 (GSK, UK), or 2′,3′-cGAMP (MCE, China) for vaccination.

Vero E6 cells (donated by Prof. Zhengli Shi from the Wuhan Institute of Virology) were maintained in Dulbecco’s modified Eagle’s medium (DMEM, Gibco, NY, USA) supplemented with 10% fetal bovine serum (FBS, Gibco, NY, USA) and cultured at 37°C with 5% CO_2_ for SARS-CoV-2 passage, titration, and neutralization. The ExpiCHO-S cells were cultured in a chemically defined serum-freeCHO medium and ExpiCHO Expression Medium (Gibco, NY, USA) for RBD protein expression.

Authentic SARS-CoV-2 (ancestral: IVCAS6.7512; Beta: IVCAS6.7552; Delta: IVCAS6.7585; Omicron BA.1: IVCAS6.7600; and Omicron XBB.1.16: IVCAS6.9083) were preserved and obtained from the National Virus Resource, Wuhan Institute of Virology, Chinese Academy of Sciences, and handled in a BSL-3 laboratory.

### Surface plasmon resonance

SPR assays were carried out using a BIAcore T200 (GE Healthcare). The ancestral, Beta, Delta, Lambda, and Omicron BA.1 RBD dimers were immobilized on CM5 chips (GE Healthcare). Human ACE2-his was diluted to 20, 10, 5, 2.5, and 1.25 nM to bind to RBD dimers. The interaction was assayed using a flow rate of 30 µL/min, an association time of 120 seconds, and a dissociation time of 300 seconds. The chip was regenerated with pH 1.5 glycine solution. The respective *K_D_* values were calculated using BIAcore T200 Evaluation 3.0 (software) with “1:1 binding” as the curve fitting method.

### Vaccination and challenge

BALB/c mice (*n* = 8 or *n* = 6) were vaccinated with AL-adjuvanted RBD dimers (10 µg per mouse) according to a homogeneous prime-boost-boost protocol with 2-week intervals. For the ELISPOT assay, the ancestral RBD-LNP mRNA vaccine (2 µg per mouse) was made as a control, which was applied according to a conventional prime-boost protocol with a 3-week interval. Blood samples were collected from the ophthalmic vein 7 or 14 days after each vaccination. Specifically, 200 µL of formulated RBD vaccine (ancestral, Beta, Delta, Lambda, or Omicron BA.1) was injected into the hind leg muscle (IM) of each mouse. Whole blood was collected from mice and stored at 4°C, and sera were separated after centrifugation at 4°C.

Syrian hamsters (*n* = 4) were vaccinated with Omicron XBB.1.5 RBD dimers (10 µg per hamster) formulated with AS03 via IM injection according to a homogeneous three-dose prime-boost-boost protocol with 2-week intervals. Syrian hamsters that underwent the vaccination program were challenged with 1 × 10^4^ PFUs of Omicron XBB.1.16 virus, and lung, nasal turbinate tissue, and trachea were obtained at 3 dpi for viral detection.

Adult hACE2-transgenic C57BL/6J mice (*n* = 6, *n* = 3, or *n* = 5) were vaccinated with RBD dimers (10 µg per mouse) formulated with AL via IM injection according to a three-dose homogeneous prime-boost-boost protocol or a two-dose homogeneous prime accompanied by one-dose heterogeneous booster protocol with 2-week intervals, or vaccinated with Omicron XBB.1.5 RBD dimers formulated with 2′, 3′-cGAMP intranasally according to a homogeneous two-dose prime-boost protocol with a 3-week interval. Serum samples were collected every 2 weeks or 7 or 10 days after the final vaccination.

Human ACE2-transgenic C57BL/6J mice that underwent the vaccination program were transferred to the A3 lab for challenge. A total of 5 × 10^4^ PFUs of Omicron BA.1 virus were diluted with DMEM and used to inoculate each animal via the nasal cavity after isoflurane anesthesia via the airway in nonlethal models, and three mice were executed for viral detection at 2 dpi. For the lethal challenge, 1 × 10^3^ PFUs of the ancestral and Delta virus, or 1 × 10^4^ PFUs of Omicron XBB.1.16 virus were used. Bodyweight and behavior were monitored during the whole experimental period. After the challenge, lung, brain, or turbinate tissues were dissected for virus detection or histopathology examination at the endpoint.

### Enzyme-linked immunosorbent assay

A 96-well polystyrene high-binding flat-bottom plate (Greiner, Germany) was coated with ancestral, Beta, Delta, Lambda, Omicron BA.1, Omicron XBB.1.5, or XBB.1.16 RBD at 1 µg/mL with a volume of 100 µL/well overnight at 4°C. The plates were blocked with 2% nonfat dried milk at room temperature for 1 hour after washing with PBST (phosphate buffer solution with 0.1% Tween-20) three times. Then, 10-fold gradient-diluted sera were added to each well, and incubation was performed at room temperature for 1 hour. Then, the plates were incubated with mouse or hamster horseradish peroxidase-conjugated secondary antibodies at room temperature for another hour after washing five times with PBST. Then, 100 µL/well of tetramethylbenzidine solution (Proteintech, China) was added after washing five times as above. Ten minutes later, 100 µL of 2 M HCl was added to each well, and the absorbance at 450 nm was measured with a Synergy H1 microplate reader (BioTek, USA). Samples with values greater than twice those in the controls were considered positive.

### Enzyme-linked immunospot assay

BALB/c mice (female, 6–8-week-old, *n* = 6) were vaccinated with AL adjuvant, ancestral RBD dimer vaccine, or ancestral RBD mRNA vaccine. The AL adjuvant and ancestral RBD dimer vaccine (10 µg/mouse) were administrated three times at 2-week intervals, and the RBD mRNA vaccine was administrated twice at a 3-week interval, as described in a previous report (46). Seven days after the last vaccination, splenocytes (5 × 10^5^/well) from each mouse were added to the IFN-γ/IL-2/IL-4/IL-10 antibody precoated plate kit (MabTech, USA) following the manufacturer’s instruction. Then, ancestral or Omicron BA.1 RBD protein was added to the wells. After incubation for 36 hours, the cells were removed, and the plates were processed in turn with a biotinylated detection antibody, followed by incubation with streptavidin and chromogenic substrate. Afterward, the liquid was poured, and the plate was washed with water to stop the color development process. The ELISPOT plate was placed on the holder, and the parameters for spot counting were adjusted. Images were captured and processed with an ImmunoSpot S6 reader (Cellular Technology Limited, USA).

### Plaque reduction neutralization test

Vero E6 cells seeded in a 24-well plate grown to monolayer confluence were used for plaque formation. Specifically, gradient-diluted sera in 200 µL DMEM were incubated with 400 PFUs of ancestral, Beta, Delta, or Omicron BA.1 and Omicron XBB.1.16 SARS-CoV-2 in 200 µL DMEM at 37°C for 1 hour. Then, 150 µL of the mixture was used to inoculate each well of a 24-well plate with two replicates, and incubation was performed for 1 hour at 37°C. Then, the infectious mixture was completely removed from the cells, and 1 mL of 0.9% methylcellulose-2% FBS-DMEM was added to each well. Four or five days later, the plate was soaked with 8% formaldehyde overnight and stained with 0.1% crystal violet. The plaques were manually counted.

### Viral load measurement in the tissues

Viral loads in the lung, brain, or nasal turbinate tissues were either measured by quantitative RT-PCR (qRT-PCR) for viral copies or plaque formation to measure the viral titers. Using a common protocol, the right lung or brain, the whole or half nasal turbinate was homogenized in 1 mL DMEM, 200 µL was used for viral RNA isolation according to the protocol of the RNeasy Mini kit (Qiagen, Germany), and the total RNA was eluted with 30 µL RNase-free water. Seven microliters of eluted product was transcribed in a 20 µL reaction system, and then 1 µL cDNA was used as a template for qRT-PCR with a HiScript II Kit (Vazyme, China). Viral copies were quantified using 1 µL of cDNA by a standard curve method on QuantStudio I (ABI, USA) with a pair of primers targeting the S gene. The standard curve was set from seven points in a 20 µL reaction system (2.35 × 10^8^ copies, 2.35 × 10^7^ copies, 2.35 × 10^6^ copies, 2.35 × 10^5^ copies, 2.35 × 10^4^ copies, 2.35 × 10^3^ copies, and 2.35 × 10^2^ copies). Samples with <2.35 × 10^2^ copies were defined as negative. The number of copies in positive samples was converted with the following equation: sample well copies × 20 × 30/7/0.2 mL/weight in grams for the lung or brain in 1 mL, and this normalized value is presented in the figure with limit of detection (LOD, 1 × 10^6^ copies/gram or mL).

For the detection of live virus in tissue, homogenized fluid was directly used for infection or 10-fold gradient dilution (LOD, 670 PFUs/gram for lung, 335 PFUs/gram for brain, and 67 PFUs/mL for nasal turbinate), and 150 µL of diluted fluid was used to inoculate Vero E6 monolayer cells seeded in 24-well plates with two replicates. The remaining steps were the same as the description of the PRNT protocol.

### Histopathology and immunofluorescence

After dissection, lung, brain, trachea, and nasal turbinate tissues were fixed in formalin for more than 1 week. Using common methods, tissue blocks underwent dehydration, and the paraffin-embedded tissue was prepared into 4 mm sections and then stained with H&E. After dehydration and sealing, the sections were stored or imaged by a microscopic imaging system (NIKON digital sight DS-FI2, Japan).

To detect virus antigens, sections were blocked and then incubated with a rabbit monoclonal antibody to SARS-CoV-2 N protein (CST, 26369S), followed by incubation with Cy3-conjugated goat anti-rabbit antibodies (Wuhan Bioqiandu Technology Co., Ltd, B100802) and DAPI (Sigma-Aldrich, D9542). Images were collected by fluorescence microscopy (Panoramic MIDI, 3D HISTECH).

### Data analysis

The data were processed using GraphPad Prism 8.0 software (CA, USA) and are presented as the mean ± standard error of the mean. The statistical analysis included unpaired *t*-tests, Mann-Whitney tests, or one-way ordinary ANOVA. The results were defined as ns*, P* > 0.05, *P < 0.05, **P < 0.01, ***P < 0.001, and ****P < 0.0001.

## Data Availability

All data associated with this study are presented in the paper or in the supplemental material. Requests for materials, data, or detailed methods should be directed to the lead contact, Xiaoyan Pan (panxy@wh.iov.cn).

## SUPPLEMENTAL MATERIAL

Supplemental materials jvi.01279-23-s0001.docx Supplemental figures.

ASM does not own the copyrights to Supplemental Material that may be linked to, or accessed through, an article. The authors have granted ASM a non-exclusive, world-wide license to publish the Supplemental Material files. Please contact the corresponding author directly for reuse.

## References

[1] Zhou P , Yang X-L , Wang X-G , Hu B , Zhang L , Zhang W , Si H-R , Zhu Y , Li B , Huang C-L , et al. . 2020. A pneumonia outbreak associated with a new coronavirus of probable bat origin. Nature 588:E6. doi:10.1038/s41586-020-2951-z 33199918 PMC9744119

[2] Hu B , Guo H , Zhou P , Shi ZL . 2021. Characteristics of SARS-CoV-2 and COVID-19. Nat Rev Microbiol 19:141–154. doi:10.1038/s41579-020-00459-7 33024307 PMC7537588

[3] Zhang L , Cui Z , Li Q , Wang B , Yu Y , Wu J , Nie J , Ding R , Wang H , Zhang Y , Liu S , Chen Z , He Y , Su X , Xu W , Huang W , Wang Y . 2021. Ten emerging SARS-CoV-2 spike variants exhibit variable infectivity, animal tropism, and antibody neutralization. Commun Biol 4:1196. doi:10.1038/s42003-021-02728-4 34645933 PMC8514557

[4] Akkız H . 2022. The biological functions and clinical significance of SARS-CoV-2 variants of corcern. Front Med (Lausanne) 9:849217. doi:10.3389/fmed.2022.849217 35669924 PMC9163346

[5] Hou YJ , Chiba S , Halfmann P , Ehre C , Kuroda M , Dinnon KH , Leist SR , Schäfer A , Nakajima N , Takahashi K , et al. . 2020. SARS-CoV-2 D614G variant exhibits efficient replication ex vivo and transmission in vivo. Science 370:1464–1468. doi:10.1126/science.abe8499 33184236 PMC7775736

[6] Plante JA , Liu Y , Liu J , Xia H , Johnson BA , Lokugamage KG , Zhang X , Muruato AE , Zou J , Fontes-Garfias CR , Mirchandani D , Scharton D , Bilello JP , Ku Z , An Z , Kalveram B , Freiberg AN , Menachery VD , Xie X , Plante KS , Weaver SC , Shi PY . 2021. Spike mutation D614G alters SARS-CoV-2 fitness. Nature 592:116–121. doi:10.1038/s41586-020-2895-3 33106671 PMC8158177

[7] Zhang L , Jackson CB , Mou H , Ojha A , Peng H , Quinlan BD , Rangarajan ES , Pan A , Vanderheiden A , Suthar MS , Li W , Izard T , Rader C , Farzan M , Choe H . 2020. SARS-CoV-2 spike-protein D614G mutation increases virion spike density and infectivity. Nat Commun 11:6013. doi:10.1038/s41467-020-19808-4 33243994 PMC7693302

[8] Alenquer M , Ferreira F , Lousa D , Valério M , Medina-Lopes M , Bergman M-L , Gonçalves J , Demengeot J , Leite RB , Lilue J , Ning Z , Penha-Gonçalves C , Soares H , Soares CM , Amorim MJ . 2021. Signatures in SARS-CoV-2 spike protein conferring escape to neutralizing antibodies. PLoS Pathog 17:e1009772. doi:10.1371/journal.ppat.1009772 34352039 PMC8341613

[9] Bayarri-Olmos R , Jarlhelt I , Johnsen LB , Hansen CB , Helgstrand C , Rose Bjelke J , Matthiesen F , Nielsen SD , Iversen KK , Ostrowski SR , Bundgaard H , Frikke-Schmidt R , Garred P , Skjoedt M-O . 2021. Functional effects of receptor-binding domain mutations of SARS-CoV-2 B.1.351 and P.1 variants. Front Immunol 12:757197. doi:10.3389/fimmu.2021.757197 34691078 PMC8529273

[10] Liu Y , Liu J , Plante KS , Plante JA , Xie X , Zhang X , Ku Z , An Z , Scharton D , Schindewolf C , Widen SG , Menachery VD , Shi PY , Weaver SC . 2022. The N501Y spike substitution enhances SARS-CoV-2 infection and transmission. Nature 602:294–299. doi:10.1038/s41586-021-04245-0 34818667 PMC8900207

[11] Aleem A , Akbar Samad AB , Vaqar S . 2023. Emerging variants of SARS-CoV-2 and novel therapeutics against coronavirus (COVID-19). StatPearls, Treasure Island (FL) ineligible companies.

[12] Chen J , Wang R , Gilby NB , Wei GW . 2022. Omicron variant (B.1.1.529): infectivity, vaccine breakthrough, and antibody resistance. J Chem Inf Model 62:412–422. doi:10.1021/acs.jcim.1c01451 34989238 PMC8751645

[13] Altmann DM , Boyton RJ . 2022. COVID-19 vaccination: the road ahead. Science 375:1127–1132. doi:10.1126/science.abn1755 35271316

[14] Jiang S , Du L , Shi Z . 2020. An emerging Coronavirus causing pneumonia outbreak in Wuhan, China: calling for developing therapeutic and prophylactic strategies. Emerg Microbes Infect 9:275–277. doi:10.1080/22221751.2020.1723441 32005086 PMC7033706

[15] Acosta-Coley I , Cervantes-Ceballos L , Tejeda-Benítez L , Sierra-Márquez L , Cabarcas-Montalvo M , García-Espiñeira M , Coronell-Rodríguez W , Arroyo-Salgado B . 2022. Vaccines platforms and COVID-19: what you need to know. Trop Dis Travel Med Vaccines 8:20. doi:10.1186/s40794-022-00176-4 35965345 PMC9537331

[16] Morens DM , Taubenberger JK , Fauci AS . 2022. Universal coronavirus vaccines - an urgent need. N Engl J Med 386:297–299. doi:10.1056/NEJMp2118468 34910863 PMC11000439

[17] Su S , Li W , Jiang S . 2022. Developing pan-beta-Coronavirus vaccines against emerging SARS-CoV-2 variants of concern. Trends Immunol 43:170–172. doi:10.1016/j.it.2022.01.009 35125310 PMC8758279

[18] Fang Z , Peng L , Filler R , Suzuki K , McNamara A , Lin Q , Renauer PA , Yang L , Menasche B , Sanchez A , Ren P , Xiong Q , Strine M , Clark P , Lin C , Ko AI , Grubaugh ND , Wilen CB , Chen S . 2022. Omicron-specific mRNA vaccination alone and as a heterologous booster against SARS-CoV-2. Nat Commun 13:3250. doi:10.1038/s41467-022-30878-4 35668119 PMC9169595

[19] Garcia-Beltran WF , St Denis KJ , Hoelzemer A , Lam EC , Nitido AD , Sheehan ML , Berrios C , Ofoman O , Chang CC , Hauser BM , Feldman J , Roederer AL , Gregory DJ , Poznansky MC , Schmidt AG , Iafrate AJ , Naranbhai V , Balazs AB . 2022. mRNA-based COVID-19 vaccine boosters induce neutralizing immunity against SARS-CoV-2 Omicron variant. Cell 185:457–466. doi:10.1016/j.cell.2021.12.033 34995482 PMC8733787

[20] Liu J , Xu K , Xing M , Zhuo Y , Guo J , Du M , Wang Q , An Y , Li J , Gao P , Wang Y , He F , Guo Y , Li M , Zhang Y , Zhang L , Gao GF , Dai L , Zhou D . 2021. Heterologous prime-boost immunizations with chimpanzee adenoviral vectors elicit potent and protective immunity against SARS-CoV-2 infection. Cell Discov 7:123. doi:10.1038/s41421-021-00360-4 34923570 PMC8684349

[21] Xu K , Gao P , Liu S , Lu S , Lei W , Zheng T , Liu X , Xie Y , Zhao Z , Guo S , et al. . 2022. Protective prototype-beta and delta-Omicron chimeric RBD-dimer vaccines against SARS-CoV-2. Cell 185:2265–2278. doi:10.1016/j.cell.2022.04.029 35568034 PMC9042943

[22] Pan X , Shi J , Hu X , Wu Y , Zeng L , Yao Y , Shang W , Liu K , Gao G , Guo W , et al. . 2021. RBD-homodimer, a COVID-19 subunit vaccine candidate, elicits immunogenicity and protection in rodents and nonhuman primates. Cell Discov 7:82. doi:10.1038/s41421-021-00320-y 34493710 PMC8423076

[23] Pan X , Zhou P , Fan T , Wu Y , Zhang J , Shi X , Shang W , Fang L , Jiang X , Shi J , Sun Y , Zhao S , Gong R , Chen Z , Xiao G . 2020. Immunoglobulin fragment F(ab')(2) against RBD potently neutralizes SARS-CoV-2 in vitro. Antiviral Res 182:104868. doi:10.1016/j.antiviral.2020.104868 32659292 PMC7351055

[24] Dai L , Zheng T , Xu K , Han Y , Xu L , Huang E , An Y , Cheng Y , Li S , Liu M , Yang M , Li Y , Cheng H , Yuan Y , Zhang W , Ke C , Wong G , Qi J , Qin C , Yan J , Gao GF . 2020. A universal design of betacoronavirus vaccines against COVID-19, MERS, and SARS. Cell 182:722–733. doi:10.1016/j.cell.2020.06.035 32645327 PMC7321023

[25] Marcotte H , Hammarström L , Pan-Hammarström Q . 2022. Limited cross-variant neutralization after primary Omicron infection: consideration for a variant-containing booster. Signal Transduct Target Ther 7:294. doi:10.1038/s41392-022-01146-0 35995763 PMC9395412

[26] Suryawanshi RK , Chen IP , Ma T , Syed AM , Brazer N , Saldhi P , Simoneau CR , Ciling A , Khalid MM , Sreekumar B , et al. . 2022. Limited cross-variant immunity from SARS-CoV-2 Omicron without vaccination. Nature 607:351–355. doi:10.1038/s41586-022-04865-0 35584773 PMC9279157

[27] Baras B , Bouveret N , Devaster J-M , Fries L , Gillard P , Sänger R , Hanon E . 2008. A vaccine manufacturer’s approach to address medical needs related to seasonal and pandemic influenza viruses. Influenza Other Respir Viruses 2:251–260. doi:10.1111/j.1750-2659.2008.00054.x 19453402 PMC2710798

[28] Wang J , Li P , Yu Y , Fu Y , Jiang H , Lu M , Sun Z , Jiang S , Lu L , Wu MX . 2020. Pulmonary surfactant-biomimetic nanoparticles potentiate heterosubtypic influenza immunity. Science 367:eaau0810. doi:10.1126/science.aau0810 32079747 PMC7432993

[29] Viana R , Moyo S , Amoako DG , Tegally H , Scheepers C , Althaus CL , Anyaneji UJ , Bester PA , Boni MF , Chand M , et al. . 2022. Rapid epidemic expansion of the SARS-CoV-2 Omicron variant in Southern Africa. Nature 603:679–686. doi:10.1038/s41586-022-04411-y 35042229 PMC8942855

[30] Yin W , Xu Y , Xu P , Cao X , Wu C , Gu C , He X , Wang X , Huang S , Yuan Q , et al. . 2022. Structures of the Omicron spike trimer with ACE2 and an anti-omicron antibody. Science 375:1048–1053. doi:10.1126/science.abn8863 35133176 PMC8939775

[31] Gilbert PB , Montefiori DC , McDermott A , Fong Y , Benkeser D , Deng W , Zhou H , Houchens CR , Martins K , Jayashankar L , et al. . 2021. Immune correlates analysis of the mRNA-1273 COVID-19 vaccine efficacy trial. medRxiv. doi:10.1101/2021.08.09.21261290 PMC9017870 34812653

[32] Hajnik RL , Plante JA , Liang Y , Alameh M-G , Tang J , Bonam SR , Zhong C , Adam A , Scharton D , Rafael GH , Liu Y , Hazell NC , Sun J , Soong L , Shi P-Y , Wang T , Walker DH , Sun J , Weissman D , Weaver SC , Plante KS , Hu H . 2022. Dual spike and nucleocapsid mRNA vaccination confer protection against SARS-CoV-2 Omicron and delta variants in preclinical models. Sci Transl Med 14:eabq1945. doi:10.1126/scitranslmed.abq1945 36103514 PMC9926941

[33] van Doremalen N , Singh M , Saturday TA , Yinda CK , Perez-Perez L , Bohler WF , Weishampel ZA , Lewis M , Schulz JE , Williamson BN , Meade-White K , Gallogly S , Okumura A , Feldmann F , Lovaglio J , Hanley PW , Shaia C , Feldmann H , de Wit E , Munster VJ , Rosenke K . 2022. SARS-CoV-2 Omicron BA.1 and BA.2 are attenuated in rhesus macaques as compared to delta. Sci Adv 8:eade1860. doi:10.1126/sciadv.ade1860 36399566 PMC9674298

[34] Hoffmann M , Krüger N , Schulz S , Cossmann A , Rocha C , Kempf A , Nehlmeier I , Graichen L , Moldenhauer A-S , Winkler MS , Lier M , Dopfer-Jablonka A , Jäck H-M , Behrens GMN , Pöhlmann S . 2022. The Omicron variant is highly resistant against antibody-mediated neutralization: implications for control of the COVID-19 pandemic. Cell 185:447–456. doi:10.1016/j.cell.2021.12.032 35026151 PMC8702401

[35] Tarke A , Coelho CH , Zhang Z , Dan JM , Yu ED , Methot N , Bloom NI , Goodwin B , Phillips E , Mallal S , Sidney J , Filaci G , Weiskopf D , da Silva Antunes R , Crotty S , Grifoni A , Sette A . 2022. SARS-CoV-2 vaccination induces immunological T cell memory able to cross-recognize variants from alpha to Omicron. Cell 185:847–859. doi:10.1016/j.cell.2022.01.015 35139340 PMC8784649

[36] Wang X , Zhao X , Song J , Wu J , Zhu Y , Li M , Cui Y , Chen Y , Yang L , Liu J , Zhu H , Jiang S , Wang P . 2022. Homologous or heterologous booster of inactivated vaccine reduces SARS-CoV-2 Omicron variant escape from neutralizing antibodies. Emerg Microbes Infect 11:477–481. doi:10.1080/22221751.2022.2030200 35034583 PMC8820826

[37] Jara A , Undurraga EA , Zubizarreta JR , González C , Pizarro A , Acevedo J , Leo K , Paredes F , Bralic T , Vergara V , Mosso M , Leon F , Parot I , Leighton P , Suárez P , Rios JC , García-Escorza H , Araos R . 2022. Effectiveness of homologous and heterologous booster doses for an inactivated SARS-CoV-2 vaccine: a large-scale prospective cohort study. Lancet Glob Health 10:e798–e806. doi:10.1016/S2214-109X(22)00112-7 35472300 PMC9034854

[38] Abbasi J . 2022. Vaccine booster dose appears to reduce Omicron hospitalizations. JAMA 327:1323. doi:10.1001/jama.2022.5156 35412578

[39] Costa Clemens SA , Weckx L , Clemens R , Almeida Mendes AV , Ramos Souza A , Silveira MBV , da Guarda SNF , de Nobrega MM , de Moraes Pinto MI , Gonzalez IGS , et al. . 2022. Heterologous versus homologous COVID-19 booster vaccination in previous recipients of two doses of Coronavac COVID-19 vaccine in Brazil (RHH-001): a phase 4, non-inferiority, single blind, randomised study. Lancet 399:521–529. doi:10.1016/S0140-6736(22)00094-0 35074136 PMC8782575

[40] Leung NHL , Cheng SMS , Cohen CA , Martín-Sánchez M , Au NYM , Luk LLH , Tsang LCH , Kwan KKH , Chaothai S , Fung LWC , Cheung AWL , Chan KCK , Li JKC , Ng YY , Kaewpreedee P , Jia JZ , Ip DKM , Poon LLM , Leung GM , Peiris JSM , Valkenburg SA , Cowling BJ . 2023. Comparative antibody and cell-mediated immune responses, reactogenicity, and efficacy of homologous and heterologous boosting with coronavac and BNT162b2 (Cobovax): an open-label, randomised trial. Lancet Microbe 4:e670–e682. doi:10.1016/S2666-5247(23)00216-1 37549680 PMC10528748

[41] Yadav T , Kumar S , Mishra G , Saxena SK . 2023. Tracking the COVID-19 vaccines: the global landscape. Hum Vaccin Immunother 19:2191577. doi:10.1080/21645515.2023.2191577 36995773 PMC10101659

[42] An Y , Li S , Jin X , Han J-B , Xu K , Xu S , Han Y , Liu C , Zheng T , Liu M , et al. . 2022. A tandem-repeat dimeric RBD protein-based COVID-19 vaccine zf2001 protects mice and nonhuman primates. Emerg Microbes Infect 11:1058–1071. doi:10.1080/22221751.2022.2056524 35311493 PMC9009945

[43] Liang JG , Su D , Song T-Z , Zeng Y , Huang W , Wu J , Xu R , Luo P , Yang X , Zhang X , Luo S , Liang Y , Li X , Huang J , Wang Q , Huang X , Xu Q , Luo M , Huang A , Luo D , Zhao C , Yang F , Han J-B , Zheng Y-T , Liang P . 2021. S-trimer, a COVID-19 subunit vaccine candidate, induces protective immunity in nonhuman primates. Nat Commun 12:1346. doi:10.1038/s41467-021-21634-1 33649323 PMC7921634

[44] Sun S , Cai Y , Song T-Z , Pu Y , Cheng L , Xu H , Sun J , Meng C , Lin Y , Huang H , et al. . 2021. Interferon-armed RBD dimer enhances the immunogenicity of RBD for sterilizing immunity against SARS-CoV-2. Cell Res 31:1222. doi:10.1038/s41422-021-00572-z 34545190 PMC8451163

[45] Ewer KJ , Barrett JR , Belij-Rammerstorfer S , Sharpe H , Makinson R , Morter R , Flaxman A , Wright D , Bellamy D , Bittaye M , et al. . 2021. T cell and antibody responses induced by a single dose of ChAdOx1 nCoV-19 (AZD1222) vaccine in a phase 1/2 clinical trial. Nat Med 27:1116. doi:10.1038/s41591-021-01363-0 34021278

[46] Zhang N-N , Li X-F , Deng Y-Q , Zhao H , Huang Y-J , Yang G , Huang W-J , Gao P , Zhou C , Zhang R-R , et al. . 2020. A thermostable mRNA vaccine against COVID-19. Cell 182:1271–1283. doi:10.1016/j.cell.2020.07.024 32795413 PMC7377714

