# Supplementary material for: Protection of the receptor binding domain (RBD) dimer against SARS-CoV-2 and its variants
Source: J Virol. 2023 Oct 16;97(11):e01279-23. doi: 10.1128/jvi.01279-23 (PMC10688353; doi:10.1128/jvi.01279-23)
Supplement: Supplemental materials — Supplemental figures. [file jvi.01279-23-s0001.docx]

Protection of the receptor binding domain (RBD) dimer against SARS-CoV-2 and its variants

Yan Wu^a#^, Jian Shi^b#^, Xiaoxue He^a#^, Jia Lu^a,c^, Xiao Gao^a,c^, Xuerui Zhu^a^, Xinlan Chen^a,c^, Man Zhang^b^, Lijuan Fang^b^, Jing Zhang^b^, Zhiming Yuan^a,c,d^, Gengfu Xiao^a,c,d^, Pengfei Zhou^b*,^ Xiaoyan Pan^a,c,d*^

Running title: Protection of the RBD dimer from SARS-CoV-2 infection

^a^State Key Laboratory of Virology, Wuhan Institute of Virology, Chinese Academy of Sciences, Wuhan, China;

^b^Wuhan YZY Biopharma Co., Ltd., Wuhan, China;

^c^University of the Chinese Academy of Sciences, Beijing, China;

^d^Center for Biosafety Mega-Science, Wuhan Institute of Virology, Chinese Academy of Sciences, Wuhan, China.

^#^These authors contributed equally to this paper.

*Correspondence: Xiaoyan Pan, [panxy@wh.iov.cn](mailto:xiaogf@wh.iov.cn); Pengfei Zhou, [pfzhou@yzybio.com](mailto:pfzhou@yzybio.com).

# Supplementary materials

##
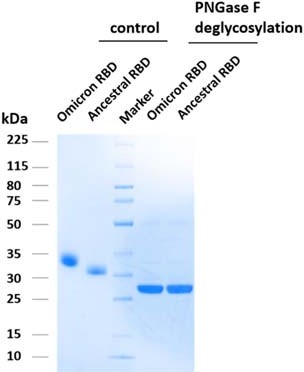
Figure s1 The deglycosylation by PNGase F. Ancestral RBD and Omicron BA.1 RBD protein were took for analysis. The picture shows that the ancestral and Omicron BA.1 RBD proteins migrated to the same position after deglycosylation by treatment with PNGase F at 37 ℃overnight, indicating that the glycosylation influences the molecular weight of the RBD proteins on SDS-PAGE.


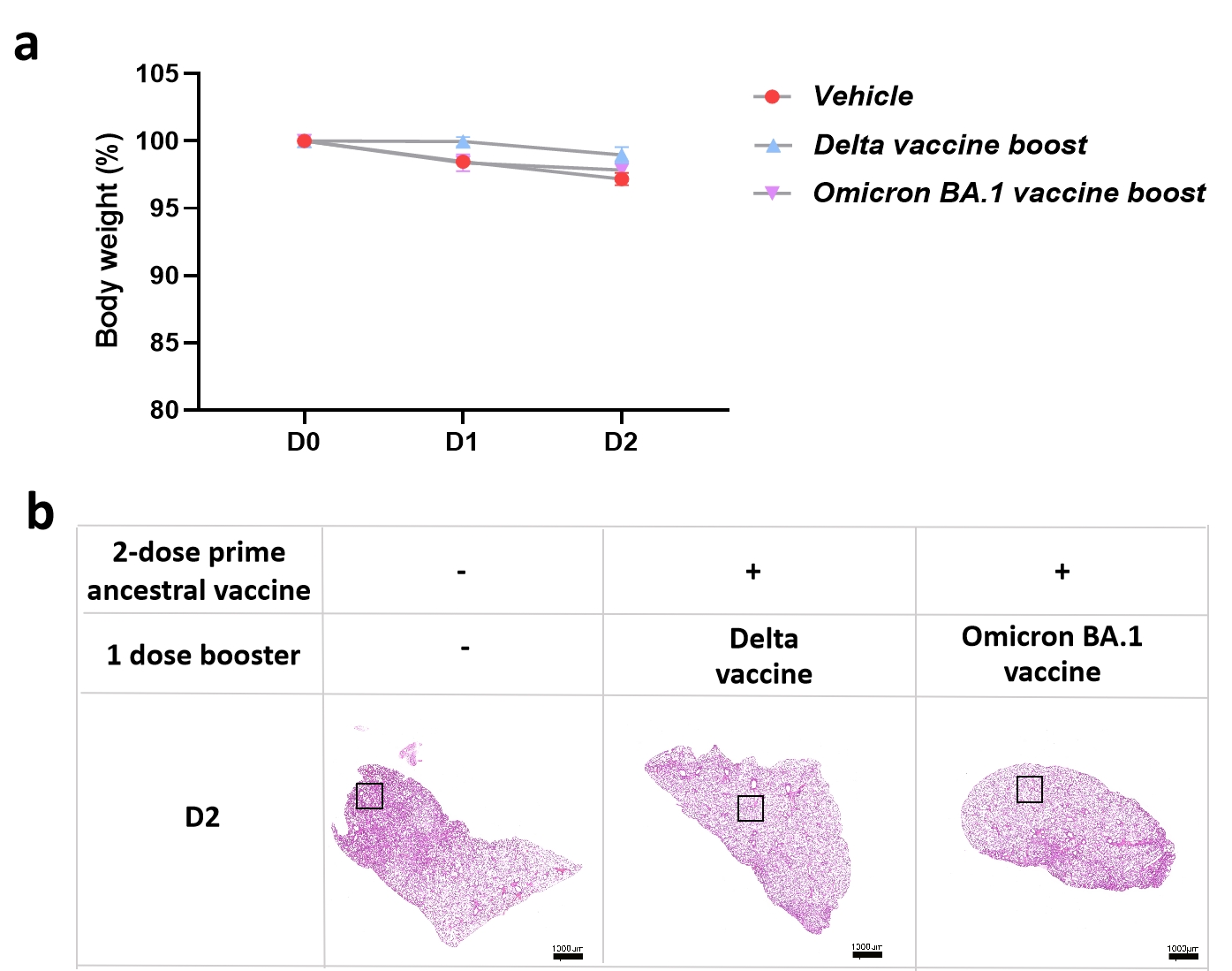


## Figure s2 The body weight change and pathological amelioration in the lung in heterogeneous booster regimen.


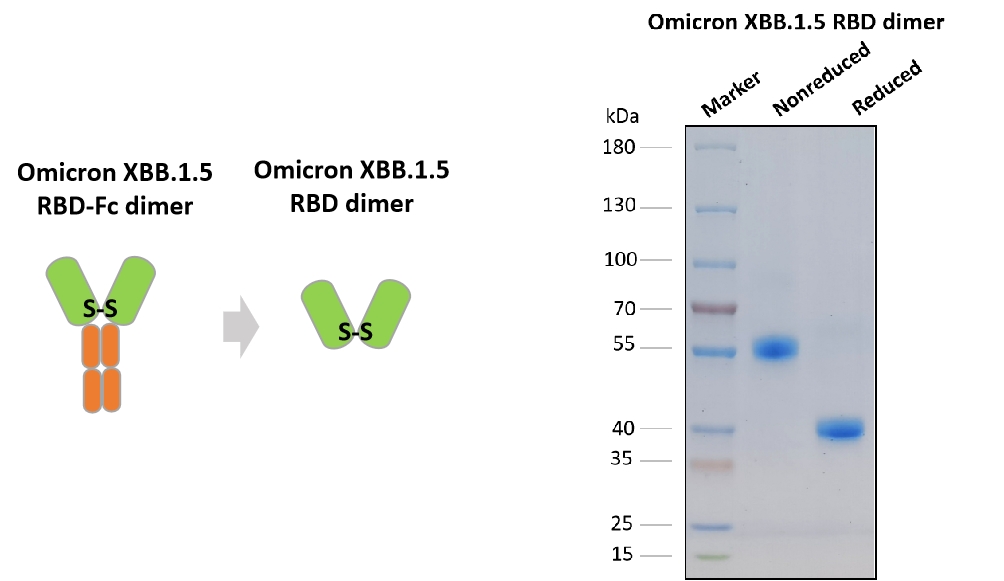


## Figure s3 The designation and characterization of SARS-CoV-2 Omicron XBB.1.5 RBD.


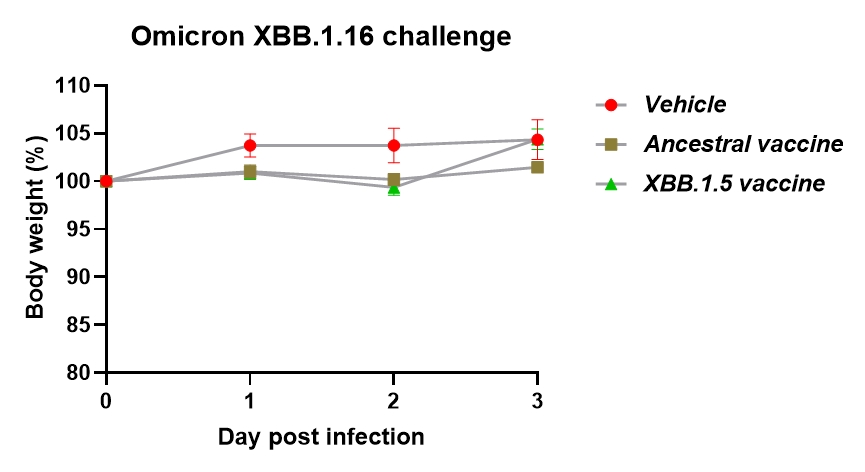


## Figure s4 The body weight change of Syrian hamster after SARS-CoV-2 XBB.1.16 challenge.
